# Relative age effects in international age group championships: A study of Spanish track and field athletes

**DOI:** 10.1371/journal.pone.0196386

**Published:** 2018-04-24

**Authors:** Javier Brazo-Sayavera, María Asunción Martínez-Valencia, Lisa Müller, Georgios Andronikos, Russell J. J. Martindale

**Affiliations:** 1 Sport Sciences Faculty, University of Extremadura, Cáceres, Spain; 2 Instituto Superior de Educación Física, Universidad de la Republica, Rivera, Uruguay; 3 Technical Area, San Ildefonso Track and Field Club, Toledo, Spain; 4 Department of Sport Science, University of Innsbruck, Innsbruck, Tyrol, Austria; 5 School of Applied Sciences, Edinburgh Napier University, Edinburgh, United Kingdom; University of Rome, ITALY

## Abstract

The relative age effect is a well-researched phenomenon, however there is still a dearth of understanding in track and field and female sport. This study investigated the role of relative age on selection for international competition of Spanish age group athletes between 2006–2014. Six hundred and forty two athletes competed for Spain at U20 or U18 age group international competition (n = 359 males; 283 females) across 9 years. The birthdates of these athletes were compared against the population of registered athletes at that time (14,502 males; 10,096 females). The results highlighted the influential role of relative age on selection to these opportunities. In line with previous research, this effect was mediated by age and gender, with stronger effects for both males and younger athletes (U18). The data best supported the ‘maturation-selection’ hypothesis as a mechanism for RAEs. These results highlight the need to carefully consider the role and need for international competitive opportunities at different age groups. A number of possible context relevant solutions are discussed, including correction adjustments techniques and competition structure within track and field.

## Introduction

As with many sports, participants in international youth track and field events are grouped by chronological age, with the aim of providing equal opportunities and competition between athletes [1]. In theory this will allow the best athletes, or those with most talent within each age band, the opportunity to shine and prove themselves on an equal footing. These competitive opportunities can potentially provide valuable learning experiences for the athletes, as well as be used to inform talent identification or selection processes. Competitive success or outcome markers of ‘talent’ (e.g. selection or medals) have also been shown to trigger subsequent confidence and commitment in athletes [2]. As such, experiences of being selected for, or performing well at international age group competitions may well be a crucial aspect of an athlete’s development, both in terms of future opportunities but also psychological development [3].

On the surface, grouping athletes in this way appears to be a practical, safe and fair method, however, in many sports research has shown that there are a disproportionate number of athletes born within the first three months of the selection year. This phenomenon is termed relative age effect (RAE) [4]. One theory that may explain the advantages that RAEs provide relatively older athletes is the maturation-selection hypothesis [4–6]. That is, chronologically older children have a higher chance of being more physically mature and therefore possessing anthropometric and physiological characteristics that aid performance (e.g., [6]). Interestingly, where RAEs exist, generally it appears to be particularly visible between the ages of 10 and 19, around the period of maturation, before becoming less prevalent, or even disappearing at adult stages [4, 7–9]. It is important to note, that there are a number of mediating factors related to the sport that can have a significant impact on the emergence of this phenomenon. For example, RAEs appear to be more prevalent for males, particularly when the demands of the sport are physical, where there is high popularity and cultural significance and when there is a strong competitive selection tier for teams, squads and competition [4, 10]. However, while research has been carried out for a number of years in this area, it is clear that there is still a dearth of evidence, particularly for female and individual sports such as track and field [4, 11].

This lack of information in this sport supports the need for continued research into RAE. From a long-term sport performance and participation perspective, RAEs provide evidence that maturity status and sport organisation can negatively impact the chance that late developers can be successful in sport, even though the link between age group success and adult elite success is poor (e.g., [12]). Indeed, the youngest athletes have been shown to be less likely to be part of representational age group teams through adolescence, and less likely to become elites [4, 13]. While RAEs are more likely to occur through adolescence, these differences could continue into elite and professional competition [14]. Furthermore, youth peers are also more likely to have poor sport experiences and drop out of sport from 14 onwards [15]. Interestingly, some evidence has shown that those youth peers who do stay in sport may be proportionally more likely to excel as adults than older peers, and have longer and more successful careers [16, 17]. As such, understanding where RAEs exist, and identifying ways to facilitate quality opportunities for all, will ultimately help both participation and adult elite sport performance, and minimise unnecessary drop out and loss of talent through the system.

In the last decade, there has been an increase in the interest of RAE research in track and field [6, 18–20]. This is perhaps unsurprising, given the sport’s popularity and figure-head status at major championships such as the Olympics [21]. Furthermore, there are a number of reasons why track and field may be particularly prone to RAEs. Track and field often uses a 2-year age range to categorise youth athletes, while other sports usually only have a 1-year age range [4]. This means those younger and probably less mature athletes are competing with an even wider range of older and likely more mature peers, widening the physical and psychological disparities further. Indeed, such a two year gap has been shown to increase RAEs [19]. Second, performance measures in track and field are based principally on physical performance measures. In this sense, results in track and field are objective [19], and crucially, it has been shown to be quite typical for coaches and scouts who search for young talent to look for physical characteristics, such as speed, power and physical size as a sign of talent [6, 22], exactly the type of characteristics influenced by previous training and maturation status.

In line with this, research within track and field has demonstrated that RAEs exist. For example, RAEs were evident at the 2008 Junior (18–19) Championships and the 2009 Youth Championships (16–17), with stronger effects at the younger age level [19]. RAE is also evident in record performances through age group athletics [23]. Furthermore, Romann and Cobley [6] investigated RAEs in Swiss age group (8–15 years old) 60m sprinters across a number of levels (local, regional, national trial event), and found evidence of RAEs across ages groups, with age and performance level acting as mediators, whereby the highest percentage of Q1 athletes occurred at the earliest (8) and latest (15) age groups, with RAEs increasing linearly with performance level. Finally, Brazo-Sayavera et al. [18] showed that RAEs existed within athletes selected for Spanish National Athletics Federation training camps at the U15 and U17 age group levels, with stronger effects for males and those athletes at the younger age level. Although some of these studies on RAE have investigated female sport (approximately 2% according to Cobley et al. [4]), this population of athletes is still significantly underrepresented. Given that gender appears to act as a mediating factor on RAEs, it seems particularly important to further research RAEs within track and field, including both males and females and not assume RAEs would apply in the same manner to both genders [24].

Recent research has identified RAEs in Spanish track and field within the talent development pathway itself, in the form of selection to national training camps [18]; however, as far as the authors are aware, there is no research investigating the relevance of RAEs at international age group competition within the Spanish track and field system. In Spain, the Spanish National Athletics Federation (RFEA) is explicit in their pursuit and support of the development of senior international track and field athletes. As such, understanding the potential influence of RAE within their age group competitive system is important, as it is highly likely that these opportunities can impact on perceptions of talent, future opportunities and decisions regarding drop out or continuation in the sport. If these systems are shown to be biased by RAEs, then many athletes with potential to be successful senior athletes may be overlooked or demoralised, resulting in a less than optimal talent development system and reduced quality at senior level [8, 11]. The aim of this study was to examine the existence of RAE by age and gender in Spanish young athletes selected to take part in international championships between 2006 and 2014.

## Materials and methods

### Participants

Date of birth of 642 athletes (359 males; 283 females) who represented Spain at international championships throughout 2006–2014 were collected from the official published lists for each championship. These lists are available at the official website of the Spanish Athletics Federation (www.rfea.es). Participants were selected from different events, such as sprints (17.4%), hurdles (10.7%), middle (10%) and long distances (8.9%), jumps (18.4%), throws (15.0%), combined events (2.6%), race walking (5.3%) and cross-country (11.7%). The authors undertook a verification of date of birth against the RFEA athletes’ database. With the purpose of comparing selected athletes with all licensed athletes in Spain, the birthdates of all athletes affiliated to the RFEA of these categories (n = 24,598) during the period studied were collected from the federation database.

### Design

This study was observational and aimed to examine RAEs, athletes’ birthdates that were categorised according to the cut-off date of 1^st^ of January [25], which is the international cut-off date for track and field. RFEA, as well IAAF, distinguishes two bi-annual age categories of competition for athletes aged between 16 and 19 years old (youth U18 and junior U20). For each of the two RFEA age categories, the athletes’ birth dates were classified into four quarters (Q1 to Q4). To test for constituent year effects, birth-dates for all athletes were analysed. Their birth year was re-coded to reflect their birth year position into year 1 for the older athletes and year 2 for the younger athletes. Given that the athletes were selected more than once, they were only added to the database the first time they were selected to take part in an international championship. Due to significant differences between gender identified in previous studies (e.g. [26]), gender was considered separately. This study was conducted in accordance with the revised ethical declaration of Helsinki and was approved by the Ethics Committee of the University of Extremadura.

### Data analysis

Frequencies were obtained for quartiles. Differences among relative age quartiles were considered using asymptotic Chi-square goodness-of-fit against an assumed equal distribution. Also, Delorme et al. [14] recommend comparing selected groups against corresponding unselected population of athletes affiliated to the RFEA, using weighted mean scores, as such this analysis was also carried out. N/A was reported when the number of athletes in a given quarter was lower than the minimum required to do the analysis.

The effect size ω for the Chi-square tests was calculated, as proposed by Wattie et al. [21]. Odds ratios (ORs) and 95% confidence intervals (95% CI) were calculated for relative age quartile distribution for the total sample and according to gender, as proposed by Cobley et al. [4]. Statistical significance was considered when p<0.05. The statistical package SPSS v.20 for MAC (IBM, New York, USA) was used for the statistical analysis; the effect size was assessed using G*Power 3.1.9.2 [27].

## Results

Table 1 presents the quarterly birth year intervals by genders, age groups and constituent years. For every category of selected athletes in the constituent year 1 significant differences were observed between the number of entries by quarter (p < 0.05). Analysing the descriptive data by individual quarters in the constituent year 1 (the older ones), it is possible to notice a higher number of athletes born in the 1st (50.6 ± 11.4%) or in 2nd quarter (35.7 ± 4.1%) of the year and very few in the last quarter (4.9 ± 6.2%). However, data from athletes in the constituent year 2 show a different distribution having in average fewer athletes born in the first quarter (18.1 ± 3.0%) than in the last quarter (26.4 ± 4.3%). Regarding unselected athletes in youth category, for both genders and constituent years, significant differences were observed between the number of entries by relative age quarter (p < 0.05). In junior category, only the male group in the constituent year 2 shows statistically significant differences by relative age quarters (p < 0.01).

**Table 1:** Relative age distribution by gender and categories of selected and corresponding unselected athletes.

				Observed frequency										
				Q1		Q2		Q3		Q4				
Category	Gender	CY	N Total	N	%	N	%	N	%	N	%	Expected frequency	χ^2^	P-value
**Selected Youth (U18)**	Male	1	61	39	63.9	21	34.4	1	1.6	0	0.0	20.3	35.514	< 0.001
		2	55	10	18.2	14	25.5	20	36.4	11	20.0	13.8	4.418	0.220
	Female	1	41	23	56.1	17	41.5	1	2.4	0	0.0	13.7	18.927	< 0.001
		2	63	14	22.2	13	20.6	18	28.6	18	28.6	15.8	1.317	0.725
**Selected Junior (U20)**	Male	1	135	57	42.2	47	34.8	22	16.3	9	6.7	33.8	43.459	< 0.001
		2	108	18	16.7	25	23.1	35	32.4	30	27.8	27.0	5.852	0.119
	Female	1	100	40	40.0	32	32.0	15	15.0	13	13.0	25.0	20.720	< 0.001
		2	79	12	15.2	24	30.4	20	25.3	23	29.1	19.8	4.494	0.213
**Unselected Youth (U18)**	Male	1	2,847	755	26.5	746	26.2	700	24.6	646	22.7	711.8	10.544	0.014
		2	8,371	2,524	30.2	2,313	27.6	1,888	22.6	1,646	19.7	2,092.8	227.449	< 0.001
	Female	1	1,351	350	25.9	376	27.8	321	23.8	304	22.5	337.8	8.979	0.030
		2	7,347	2,115	28.8	1,919	26.1	1,700	23.1	1,613	22.0	1,836.8	83.274	< 0.001
**Unselected Junior (U20)**	Male	1	1,339	334	24.9	358	26.7	328	24.5	319	23.8	334.8	2.494	0.476
		2	1,945	554	28.5	462	23.8	461	23.7	468	24.1	486.3	12.645	0.005
	Female	1	547	145	26.5	139	25.4	124	22.7	139	25.4	136.8	1.761	0.624
		2	851	235	27.6	205	24.1	208	24.4	203	23.9	212.8	3.162	0.367

CY: Constituent year. N total: Total number of participants by group. Q: Quarter.

Differences between selected and unselected athletes are displayed in Table 2. These results showed evidence of greater levels of RAE for selected athletes born in Q1 and Q2 with the consequent under-representation of athletes born in Q3 and Q4 in the first constituent year. Then, athletes in the first constituent year presented the highest over-representation in Q1 and the most distinctive under-representation in Q4, with statistically significant differences between quarters (only in junior groups). However, in the second constituent year, the two first quarters presented fewer athletes than those expected in comparison with unselected athletes distribution.

**Table 2:** Relative age distribution of selected athletes compared to corresponding unselected athletes.

			Observed frequency										
			Q1		Q2		Q3		Q4				
Category	Gender	CY	N	Δ	N	Δ	N	Δ	N	Δ	N Total	χ^2^	P-value
**Selected Youth (U18)**	Male	1	39	22,8	21	5,0	1	-14,0	0	-13,8	61	N/A	N/A
		2	10	-6,6	14	-1,2	20	7,6	11	0,2	55	7.342	0.062
	Female	1	23	12,4	17	5,6	1	-8,8	0	-9,2	41	N/A	N/A
		2	14	-4,1	13	-3,4	18	3,4	18	4,1	63	3.720	0.293
**Selected Junior (U20)**	Male	1	57	23,4	47	11,0	22	-11,1	9	-23,1	135	39.917	< 0.001
		2	18	-12,8	25	-0,7	35	9,4	30	4,0	108	9.396	0.024
	Female	1	40	13,5	32	6,6	15	-7,7	13	-12,4	100	17.258	0.001
		2	12	-9,8	24	5,0	20	0,7	23	4,1	79	6.627	0.085

CY: Constituent year. Q: Quarter. N: Number of selected athletes. Δ difference between the observed distribution and the theoretical expected distribution. N total: Total number of participants. N/A: Not applicable.

The likelihood of being selected for an international championship is described by ORs and the corresponding χ^2^ for each quarter, gender, category and constituent year in Table 3. In relation to the constituent year 1, the descriptive OR of the current findings revealed that the likelihood of being selected for an international championship is higher for an athlete of the first quarter than for one of the third quarter, including both categories. Nevertheless, the analysis of Q1:Q4 of youth athletes was not possible due to the few number of participants selected from the 4th quarter. However, in junior category, for both genders, the likelihood of being selected for an athlete of the first quarter was higher than for one of the last quarter. In addition, it was also statistically significant the OR between youth male athletes in the first and second quarters.

**Table 3:** Descriptive odds ratios (OR) and corresponding χ^2^- and p-values and effect sizes across all relative age quarters—Separated for category and constituent year.

Sample			Year 1			Year 2		
			Q1:Q2	Q1:Q3	Q1:Q4	Q1:Q2	Q1:Q3	Q1:Q4
Youth (U18)	Male [n = 116]	χ^2^	5.40	36.10	N/A	0.67	3.33	0.05
		p value	0.020	<0.001		0.414	0.068	0.827
		ω	0.3	0.96		0.16	0.34	0.04
		OR	1.86	39.0		0.71	0.5	0.91
		[95% CI]	**[1.26–2.75]**	**[5.63–270.3]**		[0.40–1.28]	[0.12–0.88]	[0.50–1.67]
	Female [n = 104]	χ^2^	0.90	20.17	N/A	0.04	0.50	0.50
		p value	0.343	<0.001		0.847	0.480	0.480
		ω	0.16	0.92		0.04	0.12	0.12
		OR	1.35	23.0		1.08	0.78	0.78
		[95% CI]	[0.86–2.12]	**[3.37–157.0]**		[0.63–1.84]	[0.47–1.28]	[0.47–1.28]
Junior (U20)	Male [n = 243]	χ^2^	0.96	15.51	34.91	1.14	5.45	3.00
		p value	0.327	<0.001	<0.001	0.286	0.020	0.083
		ω	0.1	0.44	0.72	0.16	0.32	0.24
		OR	1.21	2.59	6.33	0.72	0.51	0.6
		[95% CI]	[0.92–1.60]	**[1.77–3.79]**	**[3.43–11.71]**	[0.47–1.11]	[0.34–0.78]	[0.39–0.92]
	Female [n = 179]	χ^2^	0.89	11.36	13.76	4.00	2.00	3.46
		p value	0.346	0.001	<0.001	0.046	0.157	0.063
		ω	0.12	0.46	0.5	0.34	0.24	0.32
		OR	1.25	2.67	3.08	0.50	0.60	0.52
		[95% CI]	[0.90–1.74]	**[1.68–4.23]**	**[1.87–5.06]**	[0.30–0.84]	[0.36–1.01]	[0.31–0.88]

Bold values indicate significance of odds ratio (of 95% CI does not include 1)

Regarding the constituent year 2, there was not a statistically significant OR in any category. However, in junior male athletes, there was a statistically significant difference between the selected athletes in the first quarter in relation to those born in third quarter. In addition, in junior female athletes, it was observed a statistically difference in the number of selected athletes between first and second quarters.

The analysis of the distribution of athletes by constituent years revealed only statistically significant differences between athletes of the first and the second constituent year in youth female athletes (χ^2^ = 4.65; p = 0.031). The rest of the groups did not showed statistically significant differences, even though there were more selected athletes in the constituent year 1 than in the constituent year 2.

## Discussion

Results of the present study highlighted the existence of RAEs in Spanish athletes selected for age group international championships at U18 and U20 level. More specifically, RAE was stronger at the constituent year 1 in both categories and genders. Males as well as females in constituent year 1 showed a higher level of RAE than those selected during their constituent year 2. Previous studies have also shown the tendency of RAEs to weaken over time post maturation [4] and disappear at older age groups [18, 23, 28].

The results found in this study show a similar pattern of RAEs to work carried out in track and field by Hollings et al. [19]. Their analysis of RAEs at the 2008 Track and Field World Junior Championships (U20) and 2009 World Youth Championships (U18) found very large effects for Youth (U18) males, large effects for youth (U18) females and junior (U20) males, and moderate effects for junior (U20) females. Indeed, work investigating the role of RAEs in selection to Spanish track and field training camps, also showed similar patterns, where greater RAEs were found at younger age categories (i.e. U15) compared to the U17 category, with males showing a greater effect than females. However, interestingly, the selection to international competition in this study at a comparable age to the training camp research [18] showed a greater RAE for competition selection than training camp selection. This can be demonstrated by Q1-Q4 ORs and 95% confidence intervals (95%CI) within the two studies. However, the present study was analysed by constituent years. More specifically, male training camp OR was 2.79 (95% CI: 1.86–4.17); male competition OR was 6.98 (95% CI: 3.39–14.37); female training camp OR was 1.16 (95% CI: 0.75–1.78); and female competition OR was 2.64 (95% CI: 1.38–5.05). This evidence fits well with the hypothesis that heightened performance level, or ‘squeezed’ selection impacts on the prevalence of RAEs. For example, 1,334 athletes were selected for training camps in Brazo-Sayavera et al’s [18] study from 29,045 registered athletes (4.59%). However only 642 athletes (from an athletic population of 25,240) achieved selection to international competition in this current study, which represented 2.54% of the eligible population. This effect of increasing RAEs with performance level or more intensely selective performance groups was also shown within age group sprinting by Romann and Cobley [6].

Interestingly, when considering those registered athletes who were not selected for international competitions through the time period of 2006–2014, a relative age effect was still present in all groups, with the exception of U20 females and U20 males in the constituent year 1. One hypothesis for a lower rate of RAE in female athletes is that the sport is less popular or there are more opportunities to be selected [4]. However, in this study 14,502 (59%) male athletes do not represent the sort of disparity often seen between participation in male and female sport compared to 10,096 (41%) females athletes (e.g., [28]). Furthermore, 2.48% of male athletes from this cohort was selected for international competitions, compared with 2.8% of females. Again, suggesting that this mechanism is unlikely to be a reason for the lower levels of female RAE in this study.

The evidence in this study showing that RAE weakens across time between U18 and U20 age ranges is aligned with the maturation-selection hypothesis [4–6]. This in turn will provide performance advantages, which will likely lead to early performance success, selection and opportunities not afforded to less mature peers (e.g., [6]). This is demonstrated more directly in the literature where ‘selected’ athletes have been shown to have advanced anthropometric features, closely linked to relative age [29, 30]. Indeed, relatively younger players selected as ‘talented’ within professional English youth football programmes have been shown to be advanced physically for their age [5].

Clearly, evidence within track and field is starting to consistently reveal RAE across age groups and gender. While age and gender do act as mediators of the extent to which RAE is prevalent, it seems reasonable to conclude that this phenomenon is likely to lead to early and unnecessary drop out and reduction in the talent pool due to a bias created by maturational differences between young athletes [13]. Talented, but relatively younger athletes may not receive the opportunities or coaching standards they may need to fully develop their potential, and recreational athletes may not enjoy their experience to the full due to the bias caused through such chance factors.

Due to the potentially negative impact of RAEs on athletic development within sport, a number of ideas have been developed and tested in an attempt to reduce or eradicate RAEs, such as changing selection periods to 9 or 18 months or varying selection date [31–33]. As well, the provision of multiple squads, based on multiple standards [11, 34] or introducing quotas related to age or banding children based on maturation or weight has also been suggested and trialled [35, 36]. Less practically challenging solutions may relate to education about the existence and impact of RAEs, delaying representational sport and competition until later or de-emphasising the ‘cultural need’ to be the best at developmental stages [4, 11, 37].

However, Hollings et al. [19] have highlighted that many of these proposals would not work for international track and field age group competitions. Instead, they suggest careful planning and consideration of the timings of the staged competitions to allow all athletes the opportunity to compete at every age group competition when they are at the older end of the 2-year age group bracket. They also suggest a more radical solution, which is to take out international age group competitions at the lower age brackets (when RAE appears to be stronger) and replace them with annual regionalised competition to allow more opportunities for competition for more athletes within their regular competitive season [38]. Interestingly, developments in this regard are currently in process in the world of athletics. For example, Kenya hosted the last track and field U18 world championships in 2017, which is being discontinued. The IAAF decided it is not the best pathway for athletes at that stage of their career and that they will work with different associations to find better solutions to assist career development of these athletes [39]. Hopefully, alternative solutions will consider the role of RAEs in athletic development.

In this study the international competitions included in the analysis incorporated competitons that are held biannually (European and World Youth and Junior championships) and annually (unofficial meetings between countries and European Cross Country Championships). As such, it seems that perhaps the more radical solution suggested by Hollings and colleagues would be a more beneficial solution, particularly for males.

Furthermore, work by Romann and Cobley [6] has shown a possible solution that may be particualary suited to track and field events. They discovered that it was possible to almost completely eradicate any RAEs within age group sprinting (8–15 years old) across four different performance levels by using corrective adjustments of performance times based on relative age. Given that RAEs are likely to be a significant developmental barrier to relatively younger athletes, particulalry through their early to mid adolecent years, this could well provide an extremely useful tool to ensure that athletes are not lost, and are kept motivated and provided with appropriate opportunities through those sensitive years, when maturation has its most significant impact.

A limitation of the present study was the lack of comparison between unselected athletes and the general population. Additional information about the distribution of the general population would allow an examination of the role of RAE in young peoples’ decision to take part in track and field, and whether there is a general preference for doing track and field of those born in Q1 or Q2. However, in this research it is assumed the general population has a similar distribution. Another limitation is that the athletes were included only once in the dataset, so their first appearance in the respective list was taken throughout the period of 2006–2014: otherwise double reporting would have been possible. As such, analysis about the progression of the selected athletes through the years was not possible.

Clearly, the evidence of RAEs in selection to international age group competition in Spanish track and field is a significant finding, which has implications for the effectiveness of talent development pathways in Spain. It is clearly not a culturally specific problem and as such, research investigating any potential solutions that can have a real and applied impact on athlete experiences will be highly valuable. As it stands there is a dearth of research on the efficacy of potential solutions, so future research that investigates applied interventions would be an important avenue.
